# Oligoasthenoteratozoospermia and Infertility in Mice Deficient for miR-34b/c and miR-449 Loci

**DOI:** 10.1371/journal.pgen.1004597

**Published:** 2014-10-16

**Authors:** Stefano Comazzetto, Monica Di Giacomo, Kasper Dindler Rasmussen, Christian Much, Chiara Azzi, Emerald Perlas, Marcos Morgan, Dónal O'Carroll

**Affiliations:** European Molecular Biology Laboratory (EMBL), Mouse Biology Unit, Monterotondo Scalo, Italy; University of Cambridge, United Kingdom

## Abstract

Male fertility requires the continuous production of high quality motile spermatozoa in abundance. Alterations in all three metrics cause oligoasthenoteratozoospermia, the leading cause of human sub/infertility. Post-mitotic spermatogenesis inclusive of several meiotic stages and spermiogenesis (terminal spermatozoa differentiation) are transcriptionally inert, indicating the potential importance for the post-transcriptional microRNA (miRNA) gene-silencing pathway therein. We found the expression of miRNA generating enzyme Dicer within spermatogenesis peaks in meiosis with critical functions in spermatogenesis. In an expression screen we identified two miRNA loci of the miR-34 family (miR-34b/c and miR-449) that are specifically and highly expressed in post-mitotic male germ cells. A reduction in several miRNAs inclusive of miR-34b/c in spermatozoa has been causally associated with reduced fertility in humans. We found that deletion of both miR34b/c and miR-449 loci resulted in oligoasthenoteratozoospermia in mice. MiR-34bc/449-deficiency impairs both meiosis and the final stages of spermatozoa maturation. Analysis of miR-34bc^−/−^;449^−/−^ pachytene spermatocytes revealed a small cohort of genes deregulated that were highly enriched for miR-34 family target genes. Our results identify the miR-34 family as the first functionally important miRNAs for spermatogenesis whose deregulation is causal to oligoasthenoteratozoospermia and infertility.

## Author Summary

The sustained production of functional motile sperm is critical for male fertility. In recent years, a dramatic increase of cases of male infertility were reported, with the most common cause represented by the production of morphologically abnormal spermatozoa with low motility. Several genetic and environmental factors have been proven to impact on sperm development. In particular, preliminary studies on samples from fertile and sterile individuals suggested that the deregulation of a class of small noncoding RNAs, called microRNAs, might be detrimental for sperm formation. To this end, we investigated the expression of Dicer, a core microRNA pathway component, in male germ cells and observed a peak of expression during meiosis. We performed a microRNA-expression screening and identified 5 members of the miR-34 family (miR-34bc and miR-449abc) as highly expressed from late meiosis to the sperm stage. Deletion of miR-34bc and miR-449 leads to sterility due to the production of abnormal spermatozoa with reduced motility. Thus our work proves for the first time the importance of a microRNA family in sperm formation and male fertility.

## Introduction

Spermatogenesis is a complex developmental program that supports the generation of spermatozoa and fertility throughout the adult male life. Spermatogenesis can be divided into three principal phases, a mitotic phase, meiosis and spermiogenesis [1]. The mitotic stages of spermatogenesis encompass the spermatogonial stem cell (SSCs) as well as differentiating spermatogonia. SSCs underpin testicular homeostasis whereas the differentiating spermatogonia act as transit amplifying cells generating a large pool of cells that will undergo several terminal differentiation processes [2]. From one round of DNA replication followed by two subsequent sets of chromosomal divisions, meiosis generates round spermatids with haploid recombined genomes [3]. These round spermatids then undergo the morphogenic process of spermiogenesis that transforms these round shaped cells through an intermediate known as elongating spermatids into spermatozoa [1]. Interestingly, the meiotic stages of lepto/zygotene as well as the terminal stages of spermiogenesis are mostly transcriptionally inert suggesting the majority of the regulation of gene expression must occur at the post-transcriptional level [4], [5]. After chromosomal pairing is completed at the end of zygotene, transcription resumes in early pachytene cells [4], [5]. The full complement and importance of mechanisms that underlie the regulation of gene expression during these periods of transcriptional quiescence/reemergence remains undefined.

MiRNAs are genome encoded small 21–23 nt non-coding RNAs that negatively post-transcriptionally regulate gene expression, either through the degradation of target mRNAs or inhibition of translation [6]. MiRNAs encoding transcripts are sequentially processed by the action of two type III ribonucleases, Drosha and Dicer [7]–[10]. Drosha forms the catalytic core of the nuclear microprocessor complex that cleaves primary miRNA transcripts to yield the precursor-miRNA (pre-miR), a 60–70 nt stem loop structure [8]. Upon genesis the pre-miR is exported to the cytoplasm where it is processed by Dicer within the RNA induced silencing complex (RISC), which cleaves the terminal loop to generate an intermediate 21–22 nucleotide miRNA duplex [9]–[11]. Subsequently one strand of this duplex, the nascent miRNA, gets incorporated into an Argonaute (Ago) protein that is a key component of RISC and the execution of miRNA function [12], [13]. The miRNA defines the target specificity of RISC through base pairing with complementary RNAs [13], [14]. The majority of miRNAs display imperfect complementarity with their target transcripts and the specificity is primarily defined by the ‘seed’ (bases 2–8) sequence of the miRNA [14], [15]. MiRNA are also classified on the basis of their seed sequences, miRNA loci that share the same seed sequences are grouped into families [16]. Upon RISC binding miRNAs initially inhibit translation of the transcript followed by destabilization through the recruitment of the CCR4-NOT deadenlyation complex [17], [18]. MiRNAs exert modest changes on target gene expression ranging from 1.5–3 fold on the transcript level [19]–[21]. An interesting facet of this ubiquitous post-transcriptional gene-silencing pathway is that a given miRNA can have multiple target transcripts and thus fine-tune or buffer gene expression of numerous genes within a cell [19]–[21]. While miRNAs have been found to regulate a plethora of developmental and physiological processes, none to date have been found that regulate mammalian spermatogenesis.

## Results and Discussion

**Figure 1:**
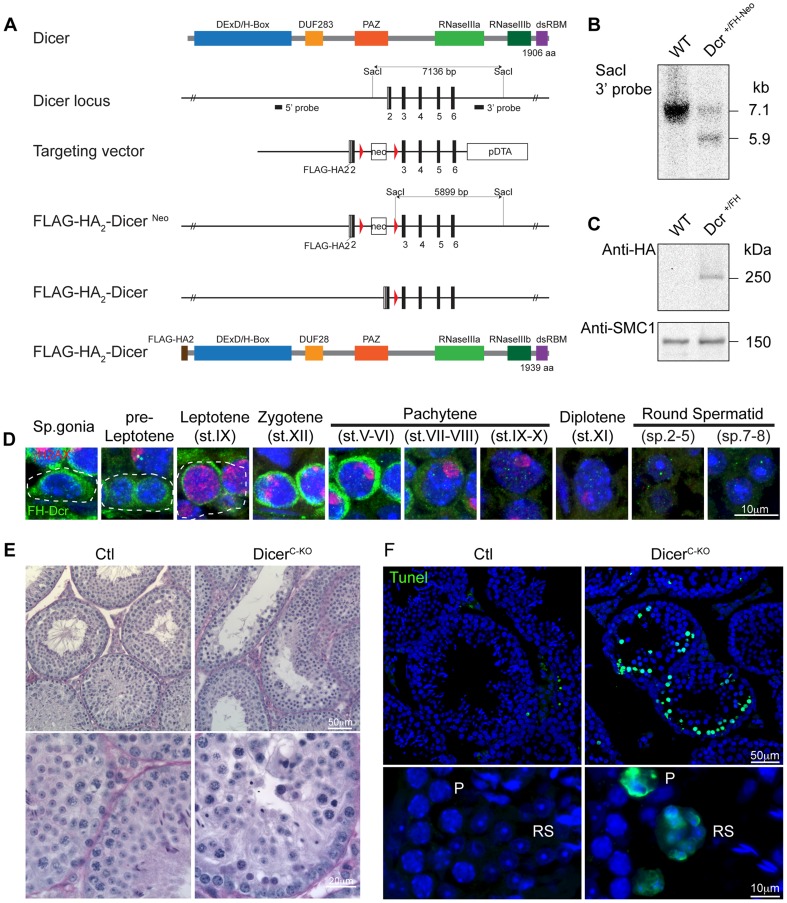
Expression and function of Dicer in adult spermatogenesis. (**A**) Domain structure of the Dicer protein is shown. The organization of the 5′ portion of *Dicer* locus is depicted. The targeting vector used for introduction of *FlagHA_2_* into the *Dicer* locus and the schematic map of the targeted *Dicer* gene before and after Cre mediated-recombination are shown. Triangles represent *loxP* sites as indicated. Rectangles indicate the position of *Neomycin (Neo)* and *Diptheria toxin A (DTA)* selection marker genes. The SacI restriction sites are indicated as well as the respective Southern fragments detected by the 3′probe. A schematic diagram of the resulting FlagHA_2_-Dicer protein is shown. (**B**) Southern blot of tail derived SacI-digested DNA from wild-type and Dcr^+/FH-Neo^ mice is shown with the 3′ probe indicated in A. (**C**) Western blot using anti-HA and anti-SMC1 antibodies on extracts from adult wild type and Dcr^+/FH^ testis is shown. (**D**) Immunofluorescence using anti-HA and anti-γH2AX antibodies on Dcr^+/FH^ testis germ cells from adult testis sections is shown. Scale bar = 10 µm. (**E**) Hematoxylin and eosin stained testis section from adult Dcr^Ctl^ and Dcr^C-KO^ mice with representative tubules shown. Scale bars = 50 µm and 20 µm in the upper and lower panel, respectively. (**F**) Increased apoptosis in Dcr^C-KO^ testis. A TUNEL assay counterstained with DAPI is shown on testis sections from adult Dcr^Ctl^ and Dcr^C-KO^ mice. The apoptotic cells stain in green. Scale bars = 50 µm and 10 µm in the upper and lower panel, respectively. Abbreviations: P, pachytene and RS, round spermatid. Representative images are shown from at least 3 mice analyzed in panels D–F.

The importance of post-transcriptional regulation of gene expression in spermatogenesis prompted us to examine the contribution of the miRNA pathway to this process. The RNase III Dicer catalyzes the last step of canonical miRNA biogenesis and thus its expression levels within testicular germ cell populations would be indicative of where this pathway or the biogenesis of miRNAs for current or later use would be important. Since antibodies against mouse Dicer that function for tissue immunofluorescence are lacking, we therefore generated a knock-in allele in mice that carry an N-terminal Flag-HA_2_ tagged Dicer (*Dcr^FH^*) (Fig. 1A–C). The Flag-HA_2_ tag did not adversely impact on the function of Dicer as mice homozygous for the *Dcr^FH^* allele are viable and fertile. Visualization in adult testis sections of Flag-HA_2_-Dicer with anti-HA antibodies revealed abundant expression of Dicer in the mitotic spermatogonia and the early meiotic stages of pre-leptotene and leptotene. Thereafter Dicer was up regulated in zygotene reaching a maximum expression in early pachytene spermatocytes (Fig. 1D). From mid-pachytene onwards Dicer was downregulated but still detected in the later stages of spermiogenesis (Fig. 1D). The expression pattern of Dicer would suggest a critical function for the miRNA pathway in meiosis as well as during haploid germ cell development. While non-canonical miRNA biogenesis pathways do exist, only a single miRNA (miR-451) has been shown to be Dicer independent [22]–[24]. In addition to miRNAs, the other Dicer products, the endogenous siRNAs, have thus far only been found in oocytes and ESCs [25]–[27]. While the failure to detect siRNAs in the male germ cells cannot formally exclude their presence therein, the loss of Dicer can more than likely be used to explore the function of the miRNA pathway in post-mitotic spermatogenesis. The importance of Dicer in early germ cell development was shown through its conditional ablation during early embryogenesis in primordial germ cells (PGCs) using the TNAP-Cre [28]. This loss of Dicer results in proliferative defects in PGCs with either absent or retarded spermatogenesis in adult seminiferous tubules [28]. To understand whether Dicer is required during meiosis, we combined the Dicer *LoxP (Dcr^Fl^)* allele with the Stra8Cre transgene that deletes in differentiating spermatogonia to generate meiotic Dicer conditional knockouts (Dicer^C-KO^) [29]–[31]. Fertility was lost in some of these animals; genotyping of pups sired by fertile Dicer^C-KO^ mice revealed the presence of the undeleted *Dcr^Fl^* allele, indicating the incomplete deletion in these animals. Histological examination of Dicer^C-KO^ testis sections revealed the presence of highly abnormal seminiferous tubules with a high apoptotic index (Fig. 1E–F). Thus the impairment of Dicer function has major impact on post-mitotic male germ cell development.

**Figure 2:**
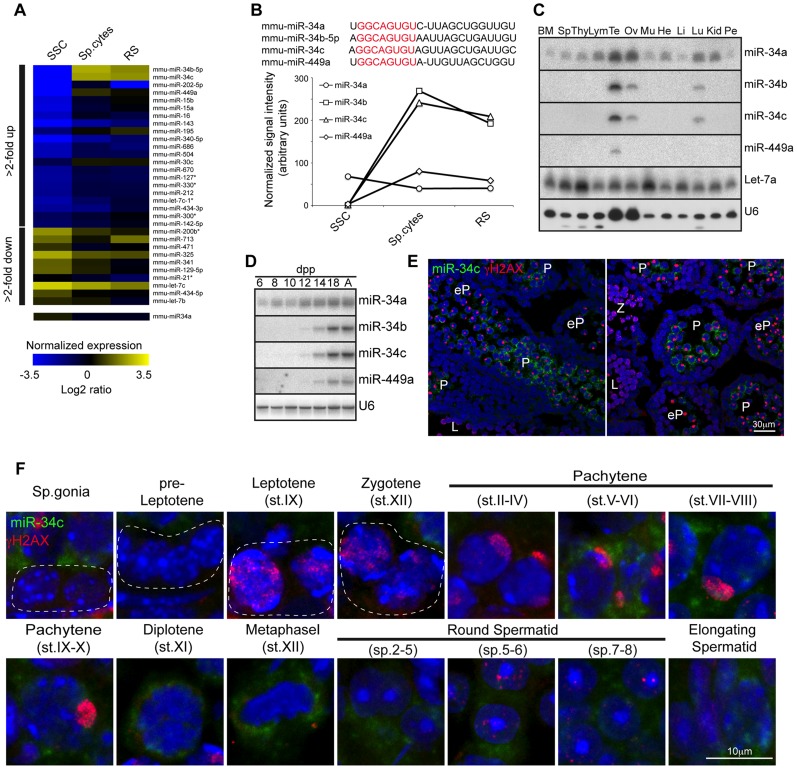
miR-34b/c and miR-449 are selectively expressed in post-mitotic spermatogenesis. (**A**) Heat diagram summarizing the expression of miRNAs in mitotic *in vitro* cultured spermatogonial stem cell (SSC) lines, *ex vivo* isolated meiotic spermatocytes (sp.cytes) and spermiogenic round spermatids (RS). The average expression of biological replicates is shown. (**B**) The mature sequence of the murine miR-34 family miRNAs is shown with the seed sequence highlighted in red. The expression of the miR-34 family members is summarized from the array data. (**C**) The expression of miR-34 family was determined by Northern blotting of RNA derived from a broad panel of tissues. U6 snRNA and Let-7a was used as loading controls. Abbreviations: BM, bone marrow; Sp, spleen; Thy, Thymus; Lym, lymph node; Te, testis; Ov, ovary; Mu, muscle, He, heart; Li, liver; Lu, lung; Kid. Kidney and Pe, peritoneal cavity cells. (**D**) Expression of miR-34 family members was determined in the first wave of spermatogenesis by Northern blotting of total RNA from testis at the indicated day post partum (dpp), Ad indicates adult. U6 snRNA was used as a loading control. Representative data is shown from two independent experiments in panel C and D. (**E**) The onset of miR-34c expression in early pachytene spermatocytes during the first wave of spermatogenesis. The spatial expression of miR-34c (Green) is shown by *in situ* hybridization on sections of 14 dpp mouse testis, the section were counterstained with anti-γH2AX (Red) antibodies to precisely identify the meiotic stage. Scale bar = 30 µm. Abbreviations: L, leptotene; Z, zygotene; eP, early pachytene and P, pachytene. (**F**) The expression of miR-34c (Green) by *in situ* hybridization in the indicated adult spermatogenic populations is shown. Anti-γH2AX (Red) was used as in (E). Scale bar = 10 µm. Representative images from one of three independent experiments are shown for panel E and F.

The expression and function of Dicer during adult spermatogenesis indicates a critical role for not just the collective miRNA pathway but also potentially for individual miRNAs. We therefore decided to perform a miRNA expression screen to identify such individual miRNA loci. To this end *in vitro* cultured SSC cell lines representative of the mitotic phase of spermatogenesis as well as *ex vivo* isolated meiotic spermatocytes and post-meiotic round spermatids were selected for miRNA profiling. Interestingly the post-mitotic spermatocyte and round spermatid populations show very similar miRNA expression profiles, that was very distinct from the mitotic SSCs with the bidirectional regulation of many miRNA loci across these developmental stages observed (Fig. 2A). MiR-34b/c stood out from this analysis due to the binary nature of their expression, essentially being absent in SSCs to representing one of the most abundantly expressed miRNAs in post mitotic germ cells (Fig. 2A–B). The miR-34b/c miRNAs are part of a miR-34 family encompassing six miRNAs (miR-34a, b, c and 449a, b, c) encoded by three distinct loci (miR-34a, miR-34b/c and miR-449) (Fig. 2B). The miR-34a locus showed ubiquitous and low levels of expression across spermatogenesis and in general ubiquitous tissue expression (Fig. 2A–C). In contrast, miR-449a displayed the binary expression as miR-34b/c during spermatogenesis but had an overall lower expression (Fig. 2A–B). Both the miR-34b/c and miR-449 showed highly restricted expression profiles across an assortment of mouse tissues (Fig. 2C) [32]. Next we wanted to determine the precise onset of miR-34b/c and miR-449a during spermatogenesis and we decided to take advantage of the first wave of spermatogenesis, as it proceeds in a near synchronous manner with the appearance of successive spermatogenic populations across juvenile mouse development (Fig. S1A). Northern blotting of testicular RNA revealed the robust expression of miR-34b/c and miR-449a at postnatal day 14, a time when the appearance of pachytene spermatocytes is observed. miRNA *in situ* coupled with immunostaining of γH2AX as a meiotic marker revealed the onset of miR-34c expression in early pachytene spermatocytes within the first wave (Fig. 2E). The same onset of expression in the adult was observed with sustained miR-34c expression detected throughout meiosis and spermiogenesis (Fig. 2F). Our analysis identifies miR-34b/c and miR-449 loci as specifically and abundantly expressed in post-mitotic germ cells.

**Figure 3:**
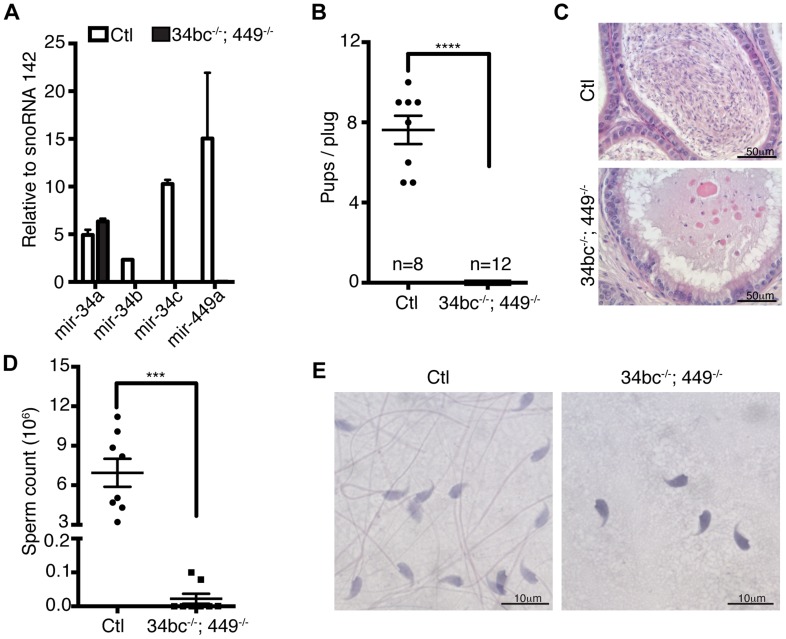
Oligoasthenoteratozoospermia and infertility in miR-34bc^−/−^;449^−/−^ mice. (**A**) qRT-PCR of miR-34a, miR-34b, miR-34c and miR-449a from control (Ctl) and miR-34bc^−/−^;449^−/−^ adult testis. (**B**) miR-34bc^−/−^;449^−/−^ male mice are infertile. The number of pups born per plug from wild type and miR-34bc^−/−^;449^−/−^ mice is shown. The number of animals tested and the mean ±s.e.m. are indicated. (**C**) Hematoxylin and eosin stained epididymis section from control (Ctl) and miR-34bc^−/−^;449^−/−^ adult mice is shown. A representative image of 5 mice analyzed is shown. Scale bar = 50 µm. (**D**) Reduced sperm count in miR-34bc^−/−^;449^−/−^ mice. Mean sperm count ±s.e.m. from control and miR-34bc^−/−^;449^−/−^ adult mice is shown (n = 8). (**E**) Sperm morphology from the indicated genotypes is shown. Scale bar = 10 µm. *** and **** indicates a *p* value (unpaired t test with Welch correction) of <0.001 and <0.0001 respectively.

The miR-34 family genes are proven important regulators of cell fate and physiology. MiR-34a and miR-34b/c loci are direct p53 target genes with the ability to repress induced reprogramming [33]–[35]. The miR34a locus also regulates cardiac function upon aging, however none of the individual miR-34 family gene disruptions affects fertility in mice (Fig. S2) [32], [34]–[36]. With the similarity of expression of miR-34b/c and miR-449 loci and their potential to be functionally redundant with respect to spermatogenesis, we generated miR-34bc^−/−^;449^−/−^ mice (Fig. 3A) that were born in Mendelian ratios. Both male and female miR-34bc^−/−^;449^−/−^ mice were infertile when mated with wild type mice (Fig. 3B). Histological analysis of epididymis revealed a dramatic reduction of spermatozoa: quantitatively this reflected in a precipitous 60-fold drop in sperm counts in the miR-34bc^−/−^;449^−/−^ mice which had an appreciable sperm count (Fig. 3C–D). Other miR-34bc^−/−^;449^−/−^ mice had so few sperm that the count approached zero (Fig. 3D). Moreover not only was a drop in quantity of mature sperm observed but also in the quality. MiR-34bc^−/−^;449^−/−^ sperm was of aberrant morphology with separation of spermatozoa heads and tails observed (Fig. 3E). Accordingly the motility of miR-34bc^−/−^;449^−/−^ sperm was severely affected. Thus miR-34bc^−/−^;449^−/−^ mice presented infertility due to low sperm count as well as spermatozoa that were immotile and of aberrant morphology. This phenotype is classified as oligoasthenoteratozoospermia and is the major cause of male infertility in humans [37].

**Figure 4:**
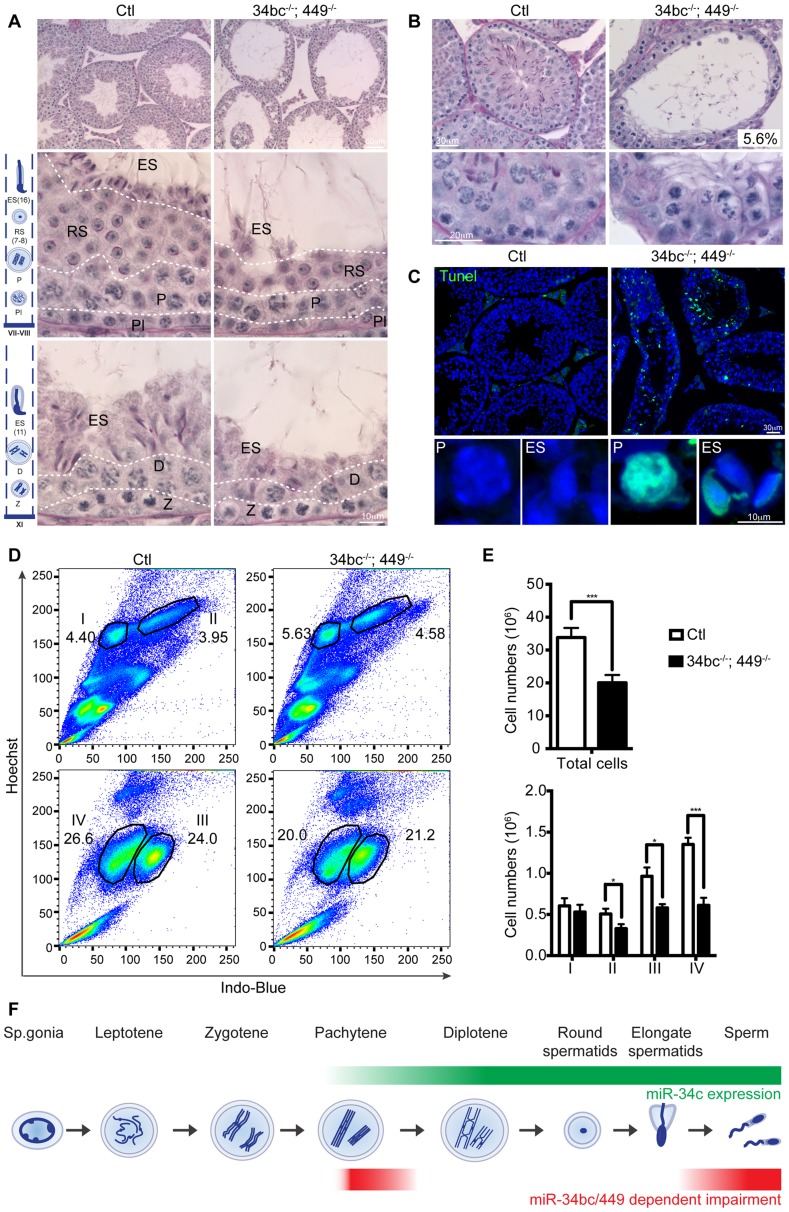
miR-34bc/449 are required for multiple stages of post-mitotic spermatogenesis. (**A**) PAS stained testis section from adult control and miR-34bc^−/−^;449^−/−^ mice is shown. Overview of several tubules is shown in upper panels. Magnified and staged tubules are presented in the lower panels, the schematic diagram summarizes the spermatogenic content of tubules in wild type mice. Abbreviations: Pl, preleptotene; Z, zygotene; P, pachytene; D, diplotene;; RS, round spermatid and ES, elongating spermatid. Scale bars = 50 µm and 10 µm in the upper and lower panels, respectively. (**B**) MiR-34bc^−/−^;449^−/−^ testis sections and percentages of tubules with meiotic arrest at zygotene stage is shown. Scale bars = 30 µm and 20 µm in the upper and lower panel, respectively. Representative images are shown from 6 mice analysed is shown in panel A and B. (**C**) Increased apoptosis in miR-34bc^−/−^;449^−/−^ testis. A TUNEL assay counterstained with DAPI (Blue) is shown on testis sections from adult control and miR-34bc^−/−^;449^−/−^ mice (upper panel). The apoptotic cells stain in green. Apoptotic miR-34bc^−/−^;449^−/−^ pachytene (P) and elongating spermatid (ES) are shown in the lower panel along with non-apoptotic control cells. Scale bars = 30 µm and 10 µm in the upper and lower panel, respectively. Representative images are shown from 3 mice analysed is shown. (**D**) FACS plot of adult testis shown, gated populations in upper panel I (lepto-zygotene) and II (pachytene-diplotene), in lower panel III (round spermatids) and IV (elongating spermatids). Numbers indicated the overall percentage of the respective populations. (**E**) Comparative enumeration of spermatogenic populations of control (Ctl) and miR-34bc/499^−/−^ mice. Total testicular cell numbers (upper) are shown. Numbers plotted for the developmentally defined subpopulations indicated in (D) by roman numerals (lower panel). 8 animals per genotype were analyzed by FACS. Mean ±s.e.m. values are shown in the graph. (**F**) Schematic diagram indicating the expression and impact of loss of miR-34bc/449 expression. * and *** indicates a *p* value (unpaired t test with Welch correction) of <0.05 and <0.001 respectively.

Having established that loss of both miR-34b/c and miR-449 loci results in oligoasthenoteratozoospermia, we next wanted to define the etiology of this disorder. To this end we studied the impact of miR-34bc/449 deficiency on spermatogenesis. Immediately obvious was the thinning of epithelium within the seminiferous tubules in miR-34bc^−/−^;449^−/−^ mice (Fig. 4A). In approximately 5% of the tubules in the testis, cells at the zygotene stage were the most advanced spermatogenic cells that could be detected (Fig. 4B). In the majority of tubules, histological examination revealed the apparently normal appearance of germ cells until the pachytene stage of development, thereafter several spermatogenic defects were observed (Fig. 4A). Specifically a reduction in the number of germ cells after pachytene stage was evident. Moreover, in miR-34bc^−/−^;449^−/−^ mice the development of the remaining round spermatids appeared to proceed normally until the final stages of spermiogenesis, when a dramatic decrease of elongating spermatids was observed (Fig. 4A). Accordingly, a high incidence of apoptosis was specifically detected in pachytene stages of meiosis as well as in elongating spermatids (Fig. 4C). The onset of the phenotype in miR-34bc^−/−^;449^−/−^ mice perfectly coincided with the expression domain of miR34b/c observed in wild type adult spermatogenesis. To precisely quantitate the impact of miR-34bc/449 deficiency on the respective germ cell populations in the testis we utilized Hoechst staining of testicular cells analyzed by FACS that can effectively discriminate between lepto-zygotene, pachytene-diplotene, round spermatid and elongating spermatid populations (Fig. 4D) [38], [39]. This analysis revealed an overall decrease in the cellularity of miR-34bc^−/−^;449^−/−^ testis but this decrease was unevenly distributed across developmental stages (Fig. 4E). Normal amounts for lepto-zygotene cells were observed in miR-34bc^−/−^;449^−/−^ testis, thereafter a significant reduction in subsequent stages was evident (Fig. 4E). Thus in combination with the histological analysis we can conclude that the miR-34 family has multiple functions during spermatogenesis both in regulating meiosis as well as the later stages of spermiogenesis (Fig. 4F).

**Figure 5:**
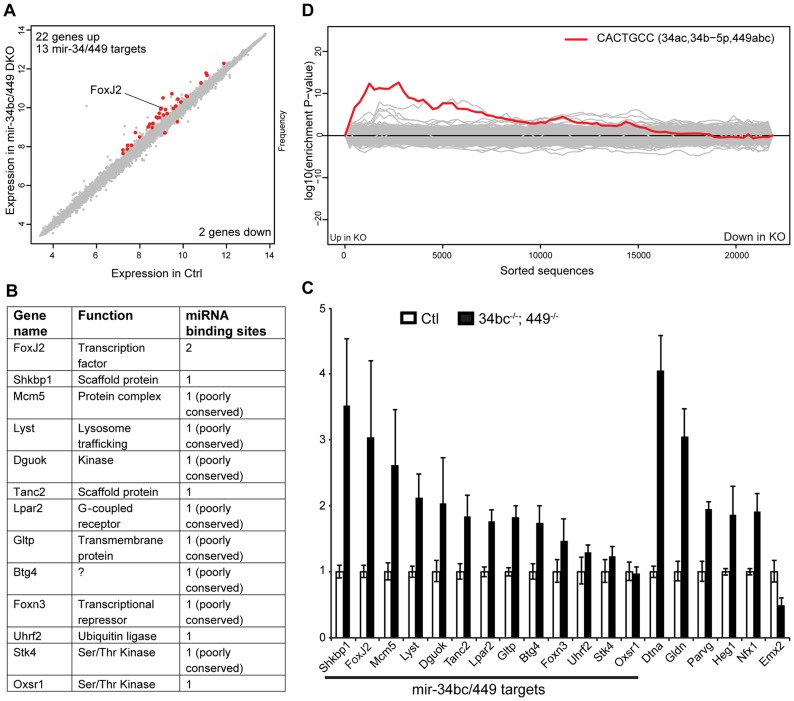
miR-34bc/449 regulates a small cohort of genes in spermatocytes. (**A**) Expression scatterplot showing relative average expression of affymetrix probes between control (x-axis) and miR-34bc^−/−^;449^−/−^ (y-axis). Significantly deregulated (p = 0.05) genes with a log_2_ fold change of 0.5 (red) are shown. (**B**) The list of the 13 upregulated genes with predicted miR-34 seed binding sites is shown. Also indicated is the gene function as well as number of miR-34 binding sites. (**C**) qRT-PCR expression analysis of representative miR-34 family seed-containing deregulated genes identified. Normalized data are plotted as relative fold change in miR-34bc^−/−^;449^−/−^ versus wild type pachytene spermatocytes. Standard error is shown and the asterisk indicates significantly upregulated expression (P<0.05). Other genes identified from the array that change in expression are also presented. The data in all panels are from biological quadruplicates of each genotype. (**D**) Sylamer enrichment landscape plot for all 876 7 nt words complementary to canonical mouse miRNA seed regions. The y-axis represents the sorted genelist of 21,560 genes from most up-regulated to most down-regulated in the miR-34bc^−/−^;449^−/−^ pachytene spermatocytes. Each 7mer word was tested for significant enrichment across the 3′UTRs of genes in this list. The word corresponding to seed matching miR-34 family (Red) is enriched in the up-regulated genes.

We next wanted to explore the mechanisms by which miR-34bc/449 supports spermatogenesis. The binding of miRNA:RISC to target mRNAs results in transcript destabilization, this facet of miRNA silencing has been used to reliably identify miRNA targets from the analysis of cellular transcriptomes with gain or loss of a specific miRNA function [19]–[21]. miRNAs exert a relatively small impact in the order of 1.5–3 fold change of target mRNAs, therefore the isolation of pure populations of cells from wild type and mutant mice is critical for comparative transcriptomic analysis and the identification of miRNA target genes. MiR-34bc/449 deficiency impacts both meiosis and the latter stages of spermiogenesis, FACS can be used to sort both of these populations, however in the case of elongating spermatids cells are damaged during the process losing both their tails and cytoplasm. We therefore decided to profile pachytene spermatocytes from control and miR-34bc^−/−^;449^−/−^ mice. This population also has the added advantage in that it is the cell type where the onset of miR-34bc/449 expression is first observed and thus likely the most promising stage to define the primary impact of miR-34bc/449-deficiency. The comparison of wild type and miR-34bc^−/−^;449^−/−^ pachytene cells revealed relatively minor changes in the transcriptome (Fig. 5A), setting a threshold of 1.4 fold (Log_2_>0.5 fold) and with significance value greater than 0.05, we found 22 genes upregulated and 2 genes downregulated in the mutant (Fig. 5A). In miRNA loss of function experiments, the expectation is the loss of repression and concomitant increase in target dosage. Strikingly, 13 of the 22 upregulated genes contained 3′UTR miR-34 ‘seed’ matches and were predicted targets of the miR-34 family (Fig. 5 A–B). Deregulation of 9 from the 13 predicted targets could be confirmed by qRT-PCR (Fig. 5C). We next employed Sylamer to search for significant enrichment of 7 nucleotide motifs corresponding to all known miRNA seed motifs across the 3′UTRs of all genes arranged from most upregulated to most downregulated in the mutant (Fig. 5D). This unbiased approach revealed a highly significant enrichment (*p* = 2.44×10^−9^) for the complementary seed match of miR-34 family (CACTGCC) in the cohort of most unregulated genes (Fig. 5D). Most importantly, no other significant miRNA seed matches were identified in this analysis. From the 9 validated miR-34 target genes identified, the forkhead transcription factor FoxJ2 merits special interest as it contains two highly conserved miR-34 binding sites and has been shown that transgenic levels of FoxJ2 overexpression are incompatible with male fertility [40]. Together, these analysis show that the loss of miR-34bc/449 has an intrinsic impact on the meiotic transcriptome and identifies a small cohort of likely direct miR-34bc/449 target genes.

Here we explored the contribution of the miRNA pathway to the post-mitotic stages of spermatogenesis. The increase in Dicer expression reaching a maximum in pachytene spermatocytes is indicative of the importance of this pathway within meiosis, indeed the loss of Dicer results in spermatogenic defects. It is interesting to note that the resumption of transcription during meiosis occurs as cells enter pachytene, therefore the high levels of Dicer therein may be required to process a burst in pre-miRNAs for current or later use. Indeed, the complement of miRNAs expressed in spermatocytes and round spermatids is very similar, thus the elevated expression of Dicer observed may be required to generate large quantities of miRNA not only for meiosis but also thereafter. In addition, the challenges presented by the resumption of transcription in pachytene cells necessitate the miRNA pathway to fine tune gene expression. This is evident from the deregulated gene expression in miR-34bc^−/−^;449^−/−^ spermatocytes and its phenotypic consequences. Our study identifies the miR-34b/c and miR-449 as the first miRNA loci required for mammalian spermatogenesis. The loss of miR-34bc/449 did not block the process of spermatogenesis *per se* but impairs several developmental transitions resulting in a low sperm count and sperm of aberrant morphology and motility. These are the phenotypic hallmarks of oligoasthenoteratozoospermia, the most common cause of reduced male fertility or infertility in humans [37]. Thus our study presents the loss of specific miRNAs as one definitive causal event in the genesis of oligoasthenoteratozoospermia. These observations bear importance for the etiology of this disorder as well as potential future basis for molecular diagnostic and therapeutic strategies.

## Materials and Methods

### Mouse strains

For the *Dcr^FH^* allele, we introduced immediately after the starting ATG of *Dicer* located within exon 2 the sequence encoding Flag-HA-HA (FlagHa_2_) epitope tags. To generate this allele, a targeting construct was generated that contains the 5′ 5.1 kb and 3′ 3.4 kb homology arms, an *loxP* flanked *neo* cassette placed in intron 2 and FlagHA_2_ sequence inserted into exon 2 as described above. Southern blotting of the individual ES cell clones-derived genomic *SacI* digested DNA with an external 3′ probe was used to identify homologous recombinants. A 7.1 kb DNA fragment corresponds to the wild-type *Dcr* locus, integration of the *neo* cassette site 5′ of exon 3 introduces an additional *SacI* site, thus decreasing the size of the *SacI* DNA fragment recognized to 5.9 kb. The integration of the FlagHA_2_ tag was confirmed by sequencing of targeted clones. Cre-mediated removal of the *loxP* flanked *neo* cassette resulted in the generation of the FlagHa_2_-Dicer (*Dcr^FH^*) allele.

The miR-34b and miR-34c miRNAs are derived from a single non-coding transcriptional unit. For the miR-34bc loss of function allele, the targeting strategy allows for Cre-mediated deletion of the hairpins that encode both miR-34b and miR-34c. To generate this allele, a targeting construct was generated that contains the 5′ 3.65 kb and 3′ 4.6 kb homology arms, an *frt* flanked *neo* cassette with a *loxP* site 5′ of the miR-34b/c encoding sequences and a second 3′ *loxP* site. Southern blotting of the individual ES cell clones-derived genomic *HindIII*-digested DNA with an external 5′ probe was used to identify homologous recombinants. A 9 kb DNA fragment corresponds to the wild-type *miR-34b/c* locus, integration of the *lox*P site 3′ of introduces an additional *HindIII* site, thus decreasing the size of the *HindIII* DNA fragment recognized to 5 kb. Flp-mediated and subsequent Cre recombination results in results in the generation *miR-34bc flox (miR-34bc^Fl^)* and *miR-34bc null (miR-34bc^−^)* alleles, respectively.

The miR-449a, miR-449b and miR-449c miRNAs are encoded in 1.6 kb of sequence within an intron of 20 Kb of the coding *Cdc20B* gene. To generate mice lacking all miR-449 miRNAs, we replaced the hairpins that encode all miR-449s with *loxP* flanked *neo* cassette. A targeting construct was generated that contains the 5′ 4.9 kb and 3′ 4.7 kb homology arms, an *loxP* flanked *neo* cassette that replaces the sequences the encoding miR-449a, b and c. Southern blotting of the individual ES cell clones-derived genomic *BamHI*-digested DNA with an external 3′ probe was used to identify homologous recombinants. A 8 kb DNA fragment corresponds to the wild-type *miR-449* locus, integration of *loxP* flanked *neo* cassette of introduces an additional *BamHI* site, thus decreasing the size of the *BamHI* DNA fragment recognized to 6.6 kb in the miR-449 targeted allele. Cre-mediated recombination removes the *neo* cassette resulting in a single scarring *loxP* site leaving the remainder of the intron *Cdc20B* intact. This strategy is designed to remove the miR-449 without affecting the *Cdc20B* gene.

The Dcr^FH^, miR-34bc and miR-449 targeting constructs were electroporated into A9 ES cells (ESCs) and manipulated to generate mice fully derived from ESCs [41]. The miR-34b/c-targeted mice were then crossed to the FLP expressing transgenic mice (FLPeR) [42] to remove the *frt* flanked *neo^r^* cassette, resulting in the generation of the *miR-34bc^Fl^* allele. Mice heterozygous for the *miR-34bc^Fl^*, targeted Dcr and miR-449 targeted alleles were crossed to Deleter Cre [43] to generate the *miR-34b^−^*, *Dcr^FH^ and miR-449^−^* alleles, respectively. Adult mice between two and four months of age on a mixed C57Bl/6 and 129 genetic background were analyzed in this study. The *Stra8-Cre* [29], [30] allele was also used in this study. For the miR-34bc^−/−^;449^−/−^ experiments, miR-34bc^+/−^;449^+/−^ or miR-34bc^+/−^ or miR-449^+/−^ were used as control mice. These control animals were normally littermates but if this was not possible age matched control mice were used. All of the mice were bred and maintained in EMBL Mouse Biology Unit, Monterotondo in accordance with current Italian legislation (Art. 9, 27. Jan 1992, n°116) under license from the Italian health ministry.

### Antibodies

A mouse monoclonal antibody against the HA epitope (Covance HA.11 Clone 16B12) was used for WB and IF (1∶1000 and 1∶100 respectively) experiments. A rabbit polyclonal anti-γH2AX (ICH, ICH-00059) (1∶250) was used for immunofluorescence and in combination with RNA *in situ* hybridization. A rabbit polyclonal anti-Smc1a (Bethyl A300-55A) (1∶10000) was used in this study.

### Immunofluorescence

Adult testes were collected and fixed in 4% paraformaldehyde overnight and embedded in paraffin. 6 µm sections were cut for HA and γH2AX immunostaining. Sections were subjected to antigen retrieval using steam vapor for 30 minutes in antigen unmasking solution (Vector Lab) and then permeabilized for 10 minutes at room temperature in 0.1% triton-X. Sections were blocked 30 minutes at room temperature in 10% normal donkey serum, 2% BSA and 0.1M glycine (Sigma). Primary antibody incubation was done overnight at 4°C in the blocking buffer. Appropriate Alexa secondary antibodies (Invitrogen) (1∶1000) were used. Hoechst 33342 (5 µg/ml) (Sigma) was used to stain DNA. Leica TCS SP5 confocal microscope was used to acquire all images. Photoshop was used for cropping and other modifications that were equally performed on control or experimental samples.

### Histology and detection of apoptotic cells

Testes were fixed in Bouin's fixative overnight at 4°C temperature, paraffin embedded and sectioned at 8-µm thickness. Sections were then stained with hematoxylin and eosin or by periodic acid Schiff and hematoxylin by using routine methods. Detection of apoptotic cells was performed on paraformaldehyde-fixed paraffin embedded testis sections using the *in situ* cell death detection kit (Roche). Sections were then stained with DAPI (5 µg/ml) (Sigma) for the identification of different germ cell populations.

### Germ cell isolation

Germ cell populations are isolated and analyzed by FACS precisely as described [38], [39], [44].

### miRNA and mRNA expression analysis

RNA was isolated using Trizol (Invitrogen) according to manufacturer's instructions. 500 ng of total RNA was labeled and hybridized to miRCURY LNA microRNA arrays V.11 (Exiqon) for miRNA profiling. Northern blotting of miRNAs was performed as described (Rasmussen, 2010). For miRNA *in situ*, testes were harvested from 4% paraformaldehyde perfused animals of various ages (6 dpp, 8 dpp, 10 dpp, 12 dpp, 14 dpp, 18 dpp, and 3 months). Tissues were further fixed by immersion in 4% paraformaldehyde overnight, cryoprotected in 30% sucrose/PBS, frozen, and sectioned at 7 µm onto Superfrost Plus slides. *In situ* hybridization was performed using LNA-probes with 3′-DIG label (Exiqon) for mir-34c. Hybridization with scramble LNA-probes were used as negative controls. Briefly, sections were digested with proteinase K for 5 min, acetylated, and hybridized with the probes in 50% formammide, 5× SSC, 5× Denhardt's solution, 500 µg/ml salmon sperm DNA, and 250 µg/ml tRNA overnight at 52.5°C. After post-hybridization washes with 50% formammide, 2× SSC at 52.5°C, and with 2× SSC at ambient temperature, sections were then blocked and incubated overnight with anti-digoxigenin-POD (Roche; at 1∶500). Signal detection was done using TSA-Plus Fluorescein system. The slides were subsequently incubated with rabbit anti-γH2AX (1∶250) and goat anti-rabbit Alexa 546 as secondary antibody. For miRNA qRT PCR, 10 ng of total RNA were reverse-transcribed using the TaqMan MicroRNA Reverse Transcription kit (4366569, Invitrogen) following manufacturer's instructions. qRT-PCRs were performed using the TaqMan Universal Master Mix II, no UNG (4440040; Invitrogen). For the reverse transcription and the qRT-PCR specific TaqMan miRNA assays (Applied Biosystem) for mir-34a, mir-34b, mir-34c, mir-449a and control snoRNA142 were used.

For mRNA microarray analysis, total RNA was isolated from FACS-sorted pachytene cells from control and mir-34bc^−/−^;449^−/−^ male mice. The RNA was hybridized to Mouse Gene 2.0 ST Arrays from Affymetrix. Data were analyzed with R/bioconductor using the limma package [45], [46]. The data was normalized and corrected from background using the Robust Multi-Array Average expression measure [47] function (rma) from the Affymetrix package. MiRNA binding motifs enrichment was analyzed using Sylamer [48].

### Accession numbers

All array data are deposited in ArrayExpress under the accession number E-MTAB-2668 and E-MTAB-2676.

## Supporting Information

Figure S1 (**A**) Schematic overview of the first wave of spermatogenesis and the days post partum (dpp) when the indicated germ cell populations are first observed. (**B**) The cellular expression of miR-34c (Green) is shown by *in situ* hybridization on sections of 14 dpp mouse testis, the section were counterstained with anti-γH2AX antibody (Red) and DAPI (blue) to precisely identify the meiotic stage. Isolated cells of the indicated stage are shown. Scale bar = 10 µm. (**C**) The expression of miR-34c (Green) as presented in (B) is shown. Staged tubules are shown. Scale bar = 10 µm. Abbreviations: B Type B spermatogonia, pL, preleptotene; L, leptotene; Z, zygotene; P, pachytene; D, diplotene; MI, metaphase I; RS, round spermatid and ES, elongating spermatid. Representative images from one of three independent experiments are shown in B–C.
(TIF)

Figure S2 (**A**) Overview of the miR-34bc locus (upper panel). Position of the DNA encoding the pre-miR-34b and pre-miR-34c are indicated. The targeting vector used for introduction of *loxP* sites into the miR-34bc locus and the schematic map of the targeted miR-34bc before and after, Flp and Cre-mediated-recombination are shown. Shaded triangles represent *lox*P sites and *frt* sites as indicated. Shaded rectangles indicate the position of Neomycin (Neo) and Diptheria toxin A (DTA) selection marker genes. The HindIII (H) restriction sites are indicated as the well as the respective Southern fragments detected by the 5′probe. (**B**) MiR-34b/c targeting diagnosed by Southern blotting of tail derived HindIII-digested DNA is presented. (**C**) The levels of miR-34b/c and other miR-34 family miRNAs in testis of the indicated genotypes determined by qRT-PCR is shown. (**D**) Overview of the miR-449 encoding region (upper panel). Position of the DNA encoding the pre-miR-449a, pre-miR-449b and pre-miR-449c are indicated within the intron of Cdc20B. The targeting vector used for introduction of *loxP* flanked Neomycin (*Neo*) cassette into the miR-449 locus and the schematic map of the targeted miR-449 before and after Cre-mediated-recombination are shown. The filled rectangles represent exons of Cdc20B. Other features are as in (A). (**E**) MiR-449 targeting diagnosed by Southern blotting of tail derived BamHI-digested DNA is presented. (**F**) The levels of miR-449a and other miR-34 family miRNAs in testis of the indicated genotypes determined by qRT-PCR is shown. (**G**) Testis weight from control (Ctl), miR-34bc^−/−^ and miR-449^−/−^ male mice is depicted. (n = 12 for controls, n = 5 for miR-34bc^−/−^ and miR-449^−/−^). (**H**) The numbers of pups per plug from control (Ctl), miR-34bc^−/−^ and miR-449^−/−^ male mice mated with wild type females is shown. (n = 10 for controls, n = 3 for miR-34bc^−/−^; n = 5 for miR-449^−/−^). (**I**) Representative images of hematoxylin and eosin stained testis sections from adult control, miR-34bc^−/−^ and miR-449^−/−^ are depicted. Scale bar = 50 µm.
(TIF)
