# Supplementary figures and images for: Oligoasthenoteratozoospermia and Infertility in Mice Deficient for miR-34b/c and miR-449 Loci
Source: PLoS Genet. 2014 Oct 16;10(10):e1004597. doi: 10.1371/journal.pgen.1004597 (PMC4199480; doi:10.1371/journal.pgen.1004597)

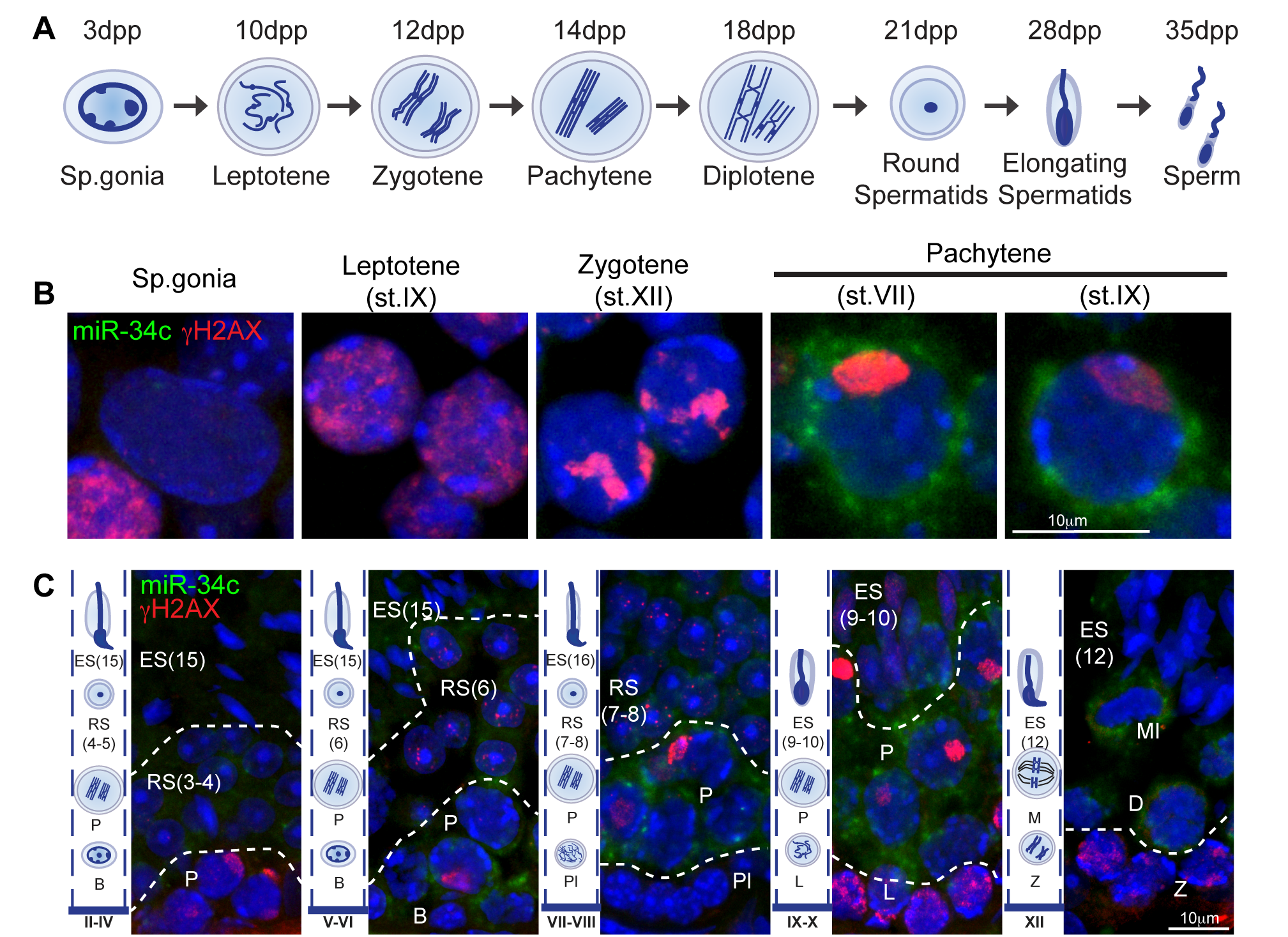

Supplement: Figure S1 — (A) Schematic overview of the first wave of spermatogenesis and the days post partum (dpp) when the indicated germ cell populations are first observed. (B) The cellular expression of miR-34c (Green) is shown by in situ hybridization on sections of 14 dpp mouse testis, the section were counterstained with anti-γH2AX antibody (Red) and DAPI (blue) to precisely identify the meiotic stage. Isolated cells of the indicated stage are shown. Scale bar = 10 µm. (C) The expression of miR-34c (Green) as presented in (B) is shown. Staged tubules are shown. Scale bar = 10 µm. Abbreviations: B Type B spermatogonia, pL, preleptotene; L, leptotene; Z, zygotene; P, pachytene; D, diplotene; MI, metaphase I; RS, round spermatid and ES, elongating spermatid. Representative images from one of three independent experiments are shown in B–C. (TIF) [file pgen.1004597.s001.tif]

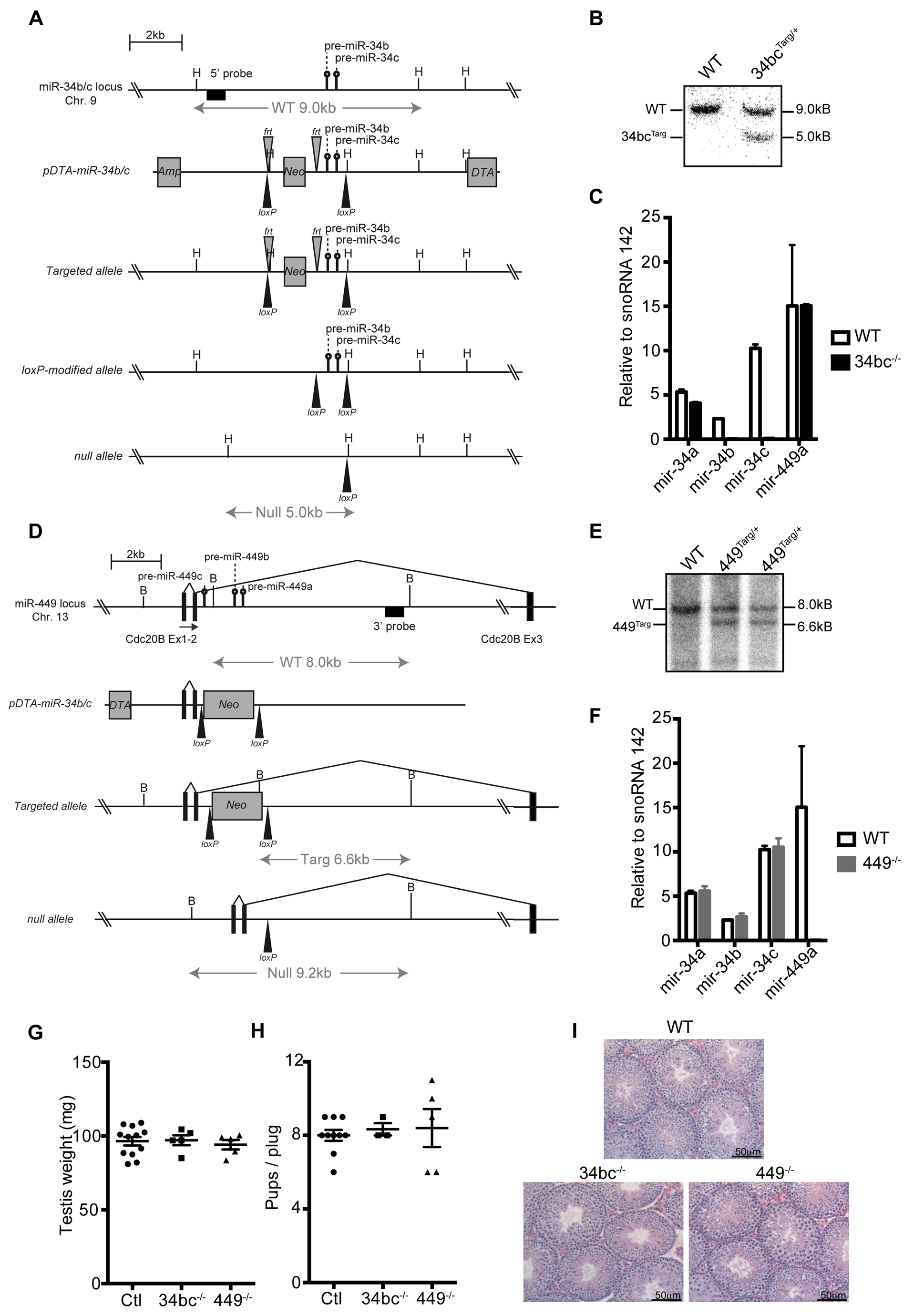

Supplement: Figure S2 — (A) Overview of the miR-34bc locus (upper panel). Position of the DNA encoding the pre-miR-34b and pre-miR-34c are indicated. The targeting vector used for introduction of loxP sites into the miR-34bc locus and the schematic map of the targeted miR-34bc before and after, Flp and Cre-mediated-recombination are shown. Shaded triangles represent loxP sites and frt sites as indicated. Shaded rectangles indicate the position of Neomycin (Neo) and Diptheria toxin A (DTA) selection marker genes. The HindIII (H) restriction sites are indicated as the well as the respective Southern fragments detected by the 5′probe. (B) MiR-34b/c targeting diagnosed by Southern blotting of tail derived HindIII-digested DNA is presented. (C) The levels of miR-34b/c and other miR-34 family miRNAs in testis of the indicated genotypes determined by qRT-PCR is shown. (D) Overview of the miR-449 encoding region (upper panel). Position of the DNA encoding the pre-miR-449a, pre-miR-449b and pre-miR-449c are indicated within the intron of Cdc20B. The targeting vector used for introduction of loxP flanked Neomycin (Neo) cassette into the miR-449 locus and the schematic map of the targeted miR-449 before and after Cre-mediated-recombination are shown. The filled rectangles represent exons of Cdc20B. Other features are as in (A). (E) MiR-449 targeting diagnosed by Southern blotting of tail derived BamHI-digested DNA is presented. (F) The levels of miR-449a and other miR-34 family miRNAs in testis of the indicated genotypes determined by qRT-PCR is shown. (G) Testis weight from control (Ctl), miR-34bc−/− and miR-449−/− male mice is depicted. (n = 12 for controls, n = 5 for miR-34bc−/− and miR-449−/−). (H) The numbers of pups per plug from control (Ctl), miR-34bc−/− and miR-449−/− male mice mated with wild type females is shown. (n = 10 for controls, n = 3 for miR-34bc−/−; n = 5 for miR-449−/−). (I) Representative images of hematoxylin and eosin stained testis sections from adult control, miR-34bc−/− and [file pgen.1004597.s002.tif]
